# 1716. Outcomes and adverse effects of risk-based perinatal antiretroviral regimens to prevent HIV transmission: A real-world experience

**DOI:** 10.1093/ofid/ofad500.1549

**Published:** 2023-11-27

**Authors:** Matty Zimmerman, Alice Hsu, Bethany Chalk, Caroline Liang, David Griffith, Nicole Salazar-Austin, Deborah Persaud, Allison Agwu

**Affiliations:** The Johns Hopkins Hospital, Baltimore, Maryland; The Johns Hopkins Hospital, Baltimore, Maryland; The Johns Hopkins Hospital, Baltimore, Maryland; Johns Hopkins Bayview Medical Center, Baltimore, Maryland; Johns Hopkins School of Medicine, Baltimore, Maryland; Johns Hopkins School of Medicine, Baltimore, Maryland; Johns Hopkins University School of Medicine, Baltimore, Maryland; Johns Hopkins University School of Medicine, Baltimore, Maryland

## Abstract

**Background:**

Annually ∼5,000 women living with HIV give birth in the US. US Public Health Guidelines recommend that perinatally-exposed infants receive antiretrovirals (ARV) to reduce transmission risk. Low risk infants receive 1 ARV (zidovudine, AZT) for 4 weeks, while highest risk infants receive 3 full dose ARVs for 6 weeks. For newborns in the moderate risk category, it is unclear what the optimal ARV regimen and duration is.

**Methods:**

We conducted a retrospective study of infants born to mothers living with HIV (MLWH) between July 2016-July 2022 at The Johns Hopkins Hospital and Bayview Medical Center. Patient demographic, medication administration, and clinical outcomes data were extracted from charts. Baseline characteristics and outcomes were summarized using descriptive statistics and stratified by risk category of the infant. Comparisons were made using the Fishers exact test for categorical data and the Kruskal Wallis test for continuous data. Statistical significance was defined as a p value < 0.05.

**Results:**

196 MLWH gave birth between July 2016 -July 2022. Mothers of high-risk infants were more likely to have lower CD4 counts, detectable viremia, illicit drug use during pregnancy, no prenatal care, and non-disclosure of their HIV status (Table 1). High-risk infants were more likely to be born at lower gestational age and have lower birth weight (Table 2). 99% of low-risk infants were prescribed AZT alone for a median of 28 days, whereas 48% of high-risk infants were prescribed 3-drug pre-emptive HIV therapy for ≥6 weeks. Amongst moderate-risk infants, 85% received 3 drug prophylaxis (nevirapine x 3 doses, lamivudine x 7 days, AZT x 6 weeks). 4 infants had a positive HIV test (Table 3), with 1 being definitively diagnosed with HIV. 3 infants had a single positive nucleic acid test that was not confirmed with additional testing.

Table 1

**Figure:**
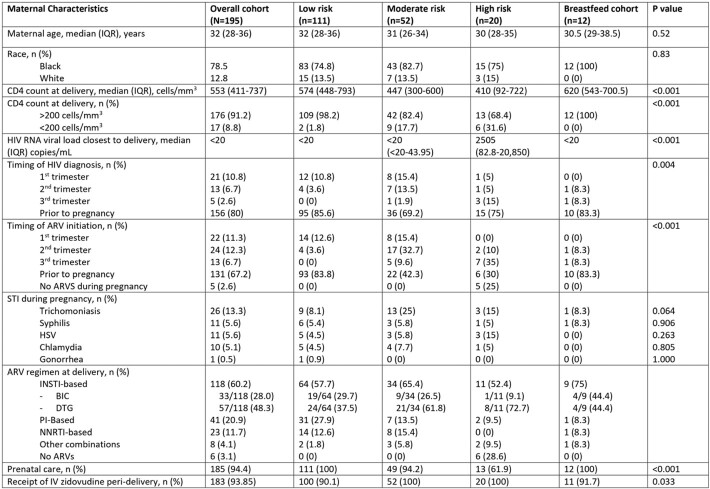

Baseline demographics of 19 5 mothers living with HIV who gave birth to infants at The Johns Hopkins Hospital or Bayview Medical Center between 2016-2022

Table 2.

**Figure:**
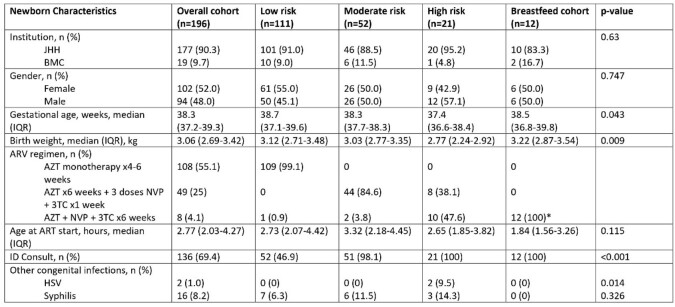

Baseline demographics of 196 infants born to MLWH at The Johns Hopkins Hospital or Bayview Medical Center between 2016-2022

Table 3

**Figure:**
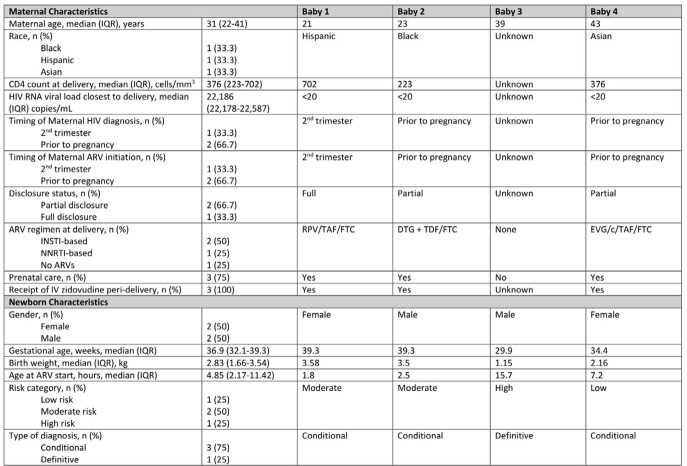

Baseline demographics of 4 infants with positive HIV tests at birth

**Conclusion:**

Amongst infants born to MLWH, various risk-based ARV regimens were prescribed and well tolerated; only 1 confirmed transmission occurred. 37% of infants were moderate/high risk requiring more extensive ARVS for longer duration and were more likely to have features which could be associated with non-adherence. These findings underscore the importance of the need for effective, discreet, shorter strategies to prevent perinatal transmission.

**Disclosures:**

**Allison Agwu, MD, ScM**, Gilead: Board Member|Gilead: Site PI for multi-center study (ongoing); Investigator initiated study|Merck: Advisor/Consultant|Merck: Site PI for multi-center study (completed)|ViiV: Board Member

